# PALOMA-3: Phase III Trial of Fulvestrant With or Without Palbociclib in Premenopausal and Postmenopausal Women With Hormone Receptor–Positive, Human Epidermal Growth Factor Receptor 2–Negative Metastatic Breast Cancer That Progressed on Prior Endocrine Therapy—Safety and Efficacy in Asian Patients

**DOI:** 10.1200/JGO.2016.008318

**Published:** 2017-04-11

**Authors:** Hiroji Iwata, Seock-Ah Im, Norikazu Masuda, Young-Hyuck Im, Kenichi Inoue, Yoshiaki Rai, Rikiya Nakamura, Jee Hyun Kim, Justin T. Hoffman, Ke Zhang, Carla Giorgetti, Shrividya Iyer, Patrick T. Schnell, Cynthia Huang Bartlett, Jungsil Ro

**Affiliations:** **Hiroji Iwata**, Aichi Cancer Center Hospital, Nagoya; **Norikazu Masuda**, National Hospital Organization, Osaka National Hospital, Osaka; **Kenichi Inoue**, Saitama Cancer Center, Saitama; **Yoshiaki Rai**, Sagara Hospital, Kagoshima City; **Rikiya Nakamura**, Chiba Cancer Center, Chiba, Japan; **Seock-Ah Im**, Seoul National University Hospital, Cancer Research Institute, Seoul National University College of Medicine; **Young-Hyuck Im**, Samsung Medical Center, Sungkyunkwan University School of Medicine, Seoul; **Jee Hyun Kim**, Seoul National University Bundang Hospital, Seongnam; **Jungsil Ro**, National Cancer Center, Goyang, Republic of Korea; **Justin T. Hoffman** and **Ke Zhang**, Pfizer, La Jolla, CA; **Carla Giorgetti**, **Shrividya Iyer**, and **Patrick T. Schnell**, Pfizer, New York, NY; and **Cynthia Huang Bartlett**, Pfizer, Collegeville, PA.

## Abstract

**Purpose:**

To assess efficacy and safety of palbociclib plus fulvestrant in Asians with endocrine therapy–resistant metastatic breast cancer.

**Patients and Methods:**

The Palbociclib Ongoing Trials in the Management of Breast Cancer 3 (PALOMA-3) trial, a double-blind phase III study, included 521 patients with hormone receptor–positive/human epidermal growth factor receptor 2–negative metastatic breast cancer with disease progression on endocrine therapy. Patient-reported outcomes (PROs) were assessed on study treatment and at the end of treatment.

**Results:**

This preplanned subgroup analysis of the PALOMA-3 study included premenopausal and postmenopausal Asians taking palbociclib plus fulvestrant (n = 71) or placebo plus fulvestrant (n = 31). Palbociclib plus fulvestrant improved progression-free survival (PFS) compared with fulvestrant alone. Median PFS was not reached with palbociclib plus fulvestrant (95% CI, 9.2 months to not reached) but was 5.8 months with placebo plus fulvestrant (95% CI, 3.5 to 9.2 months; hazard ratio, 0.485; 95% CI, 0.270 to 0.869; *P* = .0065). The most common all-cause grade 3 or 4 adverse events in the palbociclib arm were neutropenia (92%) and leukopenia (29%); febrile neutropenia occurred in 4.1% of patients. Within-patient mean trough concentration comparisons across subgroups indicated similar palbociclib exposure between Asians and non-Asians. Global quality of life was maintained; no statistically significant changes from baseline were observed for patient-reported outcome scores with palbociclib plus fulvestrant.

**Conclusion:**

This is the first report, to our knowledge, showing that palbociclib plus fulvestrant improves PFS in asian patients. Palbociclib plus fulvestrant was well tolerated in this study.

## INTRODUCTION

Breast cancer mortality rates in North American and Asian countries are comparable, with one study noting that approximately 50% to 75% of Asian women have hormone receptor (HR) –positive/human epidermal growth factor receptor 2 (HER2) –negative breast cancer.^1,2^ The median age of Asians at the time of breast cancer diagnosis (45 to 50 years) is lower than that of Western patients (55 to 60 years), including those in the United States.^3,4^ Thus, the rate of premenopausal women with breast cancer is higher in Asian populations compared with non-Asian populations.^5,6^ Cancer therapy effectiveness can also vary between Asians and non-Asians, and Asians may have a different adverse event (AE) experience versus women from other regions as a result of various reasons such as pharmacogenomics and differences in the metabolism of a specific drug.^7^

In patients with HR-positive/HER2-negative metastatic breast cancer (MBC), endocrine therapy is the mainstay of treatment^8^; however, major challenges exist when treating patients who have developed resistance to endocrine therapy with tamoxifen or aromatase inhibitors.^9,10^ Thus, treatments that can overcome endocrine therapy resistance and improve outcomes are essential.

Palbociclib, an oral small-molecule inhibitor of cyclin-dependent kinases 4 and 6 (CDK4/6), prevents DNA synthesis by blocking the progression of the cell cycle from the G_1_ to the S phase.^11,12^ The Palbociclib Ongoing Trials in the Management of Breast Cancer 3 (PALOMA-3) study included women with HR-positive/HER2-negative advanced breast cancer whose cancer had relapsed or progressed during or after prior endocrine therapy.^13,14^ In the endocrine-resistant setting, palbociclib plus fulvestrant demonstrated improved efficacy versus fulvestrant plus placebo (median progression-free survival [PFS], 9.5 *v* 4.6 months, respectively; hazard ratio [HR], 0.46; 95% CI, 0.36 to 0.59; *P* < .001).^13^ This subgroup analysis evaluates the efficacy and safety of palbociclib plus fulvestrant versus placebo plus fulvestrant in Asians and non-Asians enrolled onto PALOMA-3, a placebo-controlled clinical study.

## PATIENTS AND METHODS

### Patients and Study Design

PALOMA-3, an international, multicenter, randomized, double-blind, placebo-controlled, parallel-group, phase III clinical trial, included women with HR-positive/HER2-negative advanced breast cancer whose cancer had relapsed or progressed (on the basis of histologic or cytologic confirmation of recurrent local or distant disease progression) during or within 12 months of completing adjuvant endocrine therapy or while on or within 1 month from prior endocrine therapy for advanced breast cancer or MBC.^13,14^ One previous line of chemotherapy for advanced or metastatic disease was allowed. Asian patients in this analysis were defined as all patients who self-identified their race as Asian to investigators from the following options provided on the case report form: white, black, Asian, or other. Asian patients were included from eight study sites in Japan (n = 35), five sites in Korea (n = 43), and two sites in Taiwan (n = 4); 23 other Asian patients also were included in this analysis.

Patients were randomly assigned 2:1 to receive palbociclib plus fulvestrant or placebo plus fulvestrant. Patients received placebo or palbociclib 125 mg/d orally for 3 weeks followed by 1 week off; fulvestrant 500 mg was administered intramuscularly on days 1 and 15 of cycle 1 and then every 28 days (± 7 days) thereafter starting from day 1 of cycle 1.^13,14^ In premenopausal patients, any luteinizing hormone–releasing hormone (LHRH) agonist was administered starting at least 4 weeks before study therapy initiation. Patients who did not receive goserelin as their LHRH agonist before study entry were switched to goserelin from the time of random assignment through the entire study treatment period. The primary objective was investigator-assessed PFS; secondary objectives included clinical benefit response (CBR), objective response rate (ORR), survival probabilities, safety and tolerability, and patient-reported outcomes (PROs). In April 2015, the independent data monitoring committee reviewed the results of the study and concluded that its primary objective had been met as the study crossed the prespecified Haybittle-Peto efficacy stopping boundary (α = .00135).^13^ The updated results of the overall population have been previously published, and these data (cutoff date: March 16, 2015) were also used in this present analysis.^13^

An institutional review board/independent ethics committee approved the protocol; the study was conducted in accordance with the Declaration of Helsinki. All patients provided written informed consent before any study procedures were started. Additional patient eligibility criteria and study design details have been described previously.^13,14^

### Assessments

PFS was defined as the time from the date of random assignment to the date of first documentation of objective progression of disease or death as a result of any cause in the absence of documented progression of disease, whichever occurred first. CBR was defined as the overall rate of complete response, partial response, or stable disease ≥ 24 weeks according to the Response Evaluation Criteria in Solid Tumors (RECIST) version 1.1. Objective response was defined as the overall complete response or partial response according to RECIST version 1.1. Using x-ray, computed tomography, or magnetic resonance imaging, tumor assessments were performed at baseline and every 8 weeks for the first year and then every 12 weeks. The type, incidence, severity, and seriousness of AEs and the relationship of AEs to study medications were recorded. Severity of AEs was graded on the basis of the National Cancer Institute Common Terminology Criteria for Adverse Events version 4.0. A serious AE was defined as an AE that results in death, is life threatening, requires inpatient hospitalization or prolongation of existing hospitalization, results in persistent or significant disability or incapacity, or results in congenital anomaly or birth defect. An AE could additionally be considered serious by the investigator if it jeopardized the patient or required intervention to prevent one of the other AE outcomes.

In addition, pharmacokinetic (PK) data and PROs were assessed by race. Trough PK samples for determination of palbociclib plasma concentrations were collected from all randomly assigned patients on day 15 of cycles 1 and 2. PROs were assessed using the European Organisation for Research and Treatment of Cancer Quality of Life Questionnaire C30, a 30-item questionnaire that includes functional scales, symptom scales, and a global health status/quality-of-life (QOL) scale.^15,16^ For functional and global QOL scales, higher scores represent a better level of functioning. For symptom-oriented scales, a higher score represents more severe symptoms. PRO questionnaires were completed before dose on day 1 of cycles 1 to 4, then on day 1 of every other subsequent cycle starting with cycle 6, and finally, at the end of treatment. For PK assessments, a post hoc analysis was used for the comparison of racial subgroups.

### Statistical Analyses

Study assessments of efficacy, safety, and PROs were prespecified; efficacy subgroup analyses by various baseline variables, including race, were preplanned in the protocol and statistical analysis plan. Statistical analyses by race were conducted for exploratory purposes. Demographic and baseline disease characteristics were summarized by treatment arm in a frequency table for Asians and non-Asians. Quantitative baseline variables, including age, weight, and height, were summarized using descriptive statistics (ie, median and range). Quantitative baseline variables were compared between the two treatment arms using a Wilcoxon two-sample test without adjusting for multiplicity. Efficacy analyses were performed using the intent-to-treat principle. Kaplan-Meier estimates of median PFS and the respective 95% CIs were provided for both treatment groups. PFS data between the treatment groups were compared using a log-rank test. HR was estimated from the Cox proportional hazards regression model. The odds ratio estimator and the exact test were used to compare the rates of binary efficacy end points. AEs were summarized using descriptive statistics in Asians who took one or more doses of study treatment. The within-patient averages of the palbociclib steady-state trough PK samples were summarized and compared across subgroups. PRO analyses were based on the PRO-evaluable population (ie, patients in the intent-to-treat population with a baseline assessment and one or more postbaseline assessments before the end of study treatment). Completion rates were summarized by cycle. Repeated-measures mixed-effects analyses were performed to compare on-treatment overall scores and changes from baseline between treatment groups while controlling for baseline.

## RESULTS

### Patients

**Fig 1:**
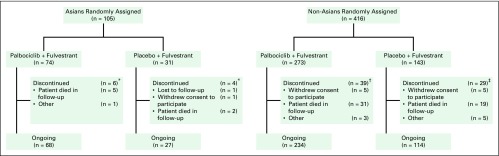
Patient disposition. (*) None of the patients discontinued treatment because of adverse events (AEs). (†) One patient (0.4%) discontinued treatment because of AEs. (‡) Two patients (1.4%) discontinued treatment because of AEs.

From October 7, 2013, to August 6, 2014, 105 Asians were enrolled onto the study (74 and 31 patients in the palbociclib and placebo arms, respectively; Fig 1). Demographic and baseline disease characteristics were generally similar between Asians and non-Asians except for age, weight, and percentage of premenopausal or perimenopausal patients. Asians, compared with non-Asians, were generally younger (mean age, 53.7 *v* 57.7 years, respectively; *P* = .0013) and weighed less (mean, 56.7 *v* 74.6 kg, respectively; *P* < .001; Table 1). The percentage of premenopausal or perimenopausal women at baseline was higher in Asians (42%) compared with non-Asians (15%). Among Asians, demographic and baseline disease characteristics were generally similar between the palbociclib and placebo arms.

**Table 1:**
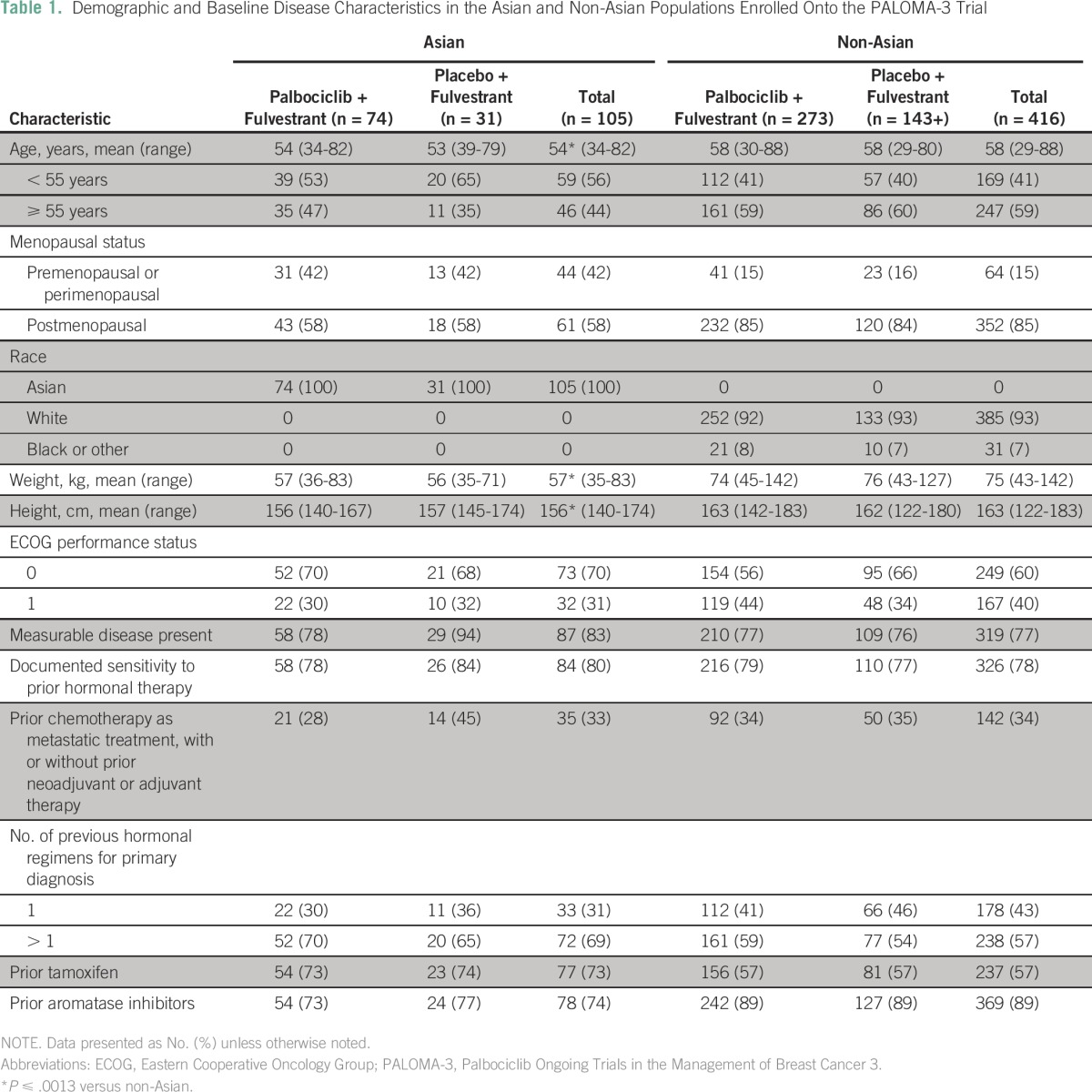
Demographic and Baseline Disease Characteristics in the Asian and Non-Asian Populations Enrolled Onto the PALOMA-3 Trial

### Efficacy

**Fig 2:**
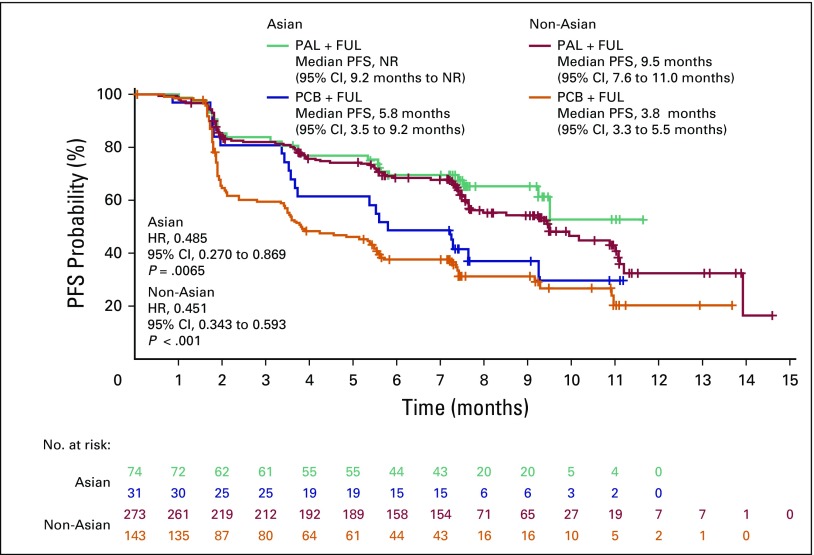
Investigator-assessed progression-free survival (PFS) in Asian and non-Asian patients. FUL, fulvestrant; HR, hazard ratio; NR, not reached; PAL, palbociclib; PCB, placebo.

The degree of PFS improvement in the palbociclib arm versus the placebo arm was similar in Asians and non-Asians (Fig 2). The median PFS in Asians was not reached in the palbociclib arm (95% CI, 9.2 months to not reached) but was 5.8 months (95% CI, 3.5 to 9.2 months) in the placebo arm (HR, 0.485; 95% CI, 0.27 to 0.87; *P* = .0065). In non-Asians, the median PFS was 9.5 months (95% CI, 7.6 to 11 months) in the palbociclib arm compared with 3.8 months (95% CI, 3.3 to 5.5 months) in the placebo arm (HR, 0.451; 95% CI, 0.34 to 0.59; *P* < .001). In Asians, the CBR was 70% (95% CI, 59% to 80%) with palbociclib plus fulvestrant and 52% (95% CI, 33% to 70%) with placebo plus fulvestrant (odds ratio, 2.216; 95% CI, 0.85 to 5.7; Table 2). In non-Asians, the CBR was 66% (95% CI, 60% to 71%) and 37% (95% CI, 29% to 46%) in the palbociclib and placebo arms, respectively (odds ratio, 3.234; 95% CI, 2.1 to 5.0; *P* < .001). The ORR in Asians was 19% in the palbociclib arm and 13% in the placebo arm. The sample size was underpowered to perform any statistical analysis. However, the degrees of improvement for CBR and ORR in Asians were similar to those seen in non-Asians.

**Table 2:**
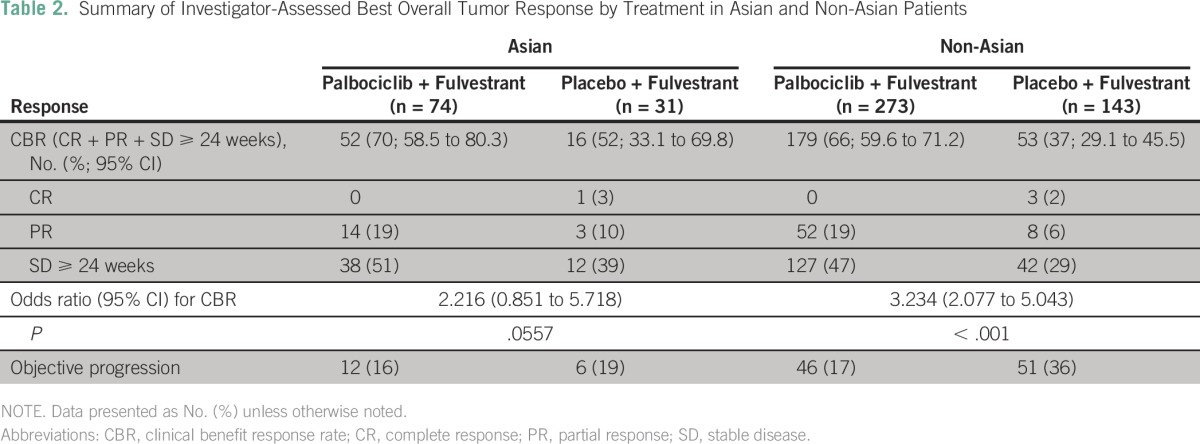
Summary of Investigator-Assessed Best Overall Tumor Response by Treatment in Asian and Non-Asian Patients

### Safety

The exposure to study treatments was comparable between Asians and non-Asians (Table 3). Among Asians, 100% of patients in the palbociclib arm and 94% in the placebo arm experienced treatment-emergent AEs of any grade (Table 4). The most common AEs among Asians were neutropenia and leukopenia. Febrile neutropenia occurred in three Asians (4%) in the palbociclib arm, with two of these cases reported as a serious AE. On the basis of results that were unadjusted for sample size differences between Asians and non-Asians, non-Asians in the palbociclib arm generally experienced similar treatment-emergent AEs at comparable incidences (< 10%); however, in Asians, compared with non-Asians, the incidence of fatigue (19% *v* 44%, respectively) was lower, and the rates of neutropenia (92% *v* 78%, respectively), stomatitis (41% *v* 24%, respectively), rash (32% *v* 11%, respectively), and nasopharyngitis (21% *v* 10%, respectively) were higher (Table 4).

**Table 3:**
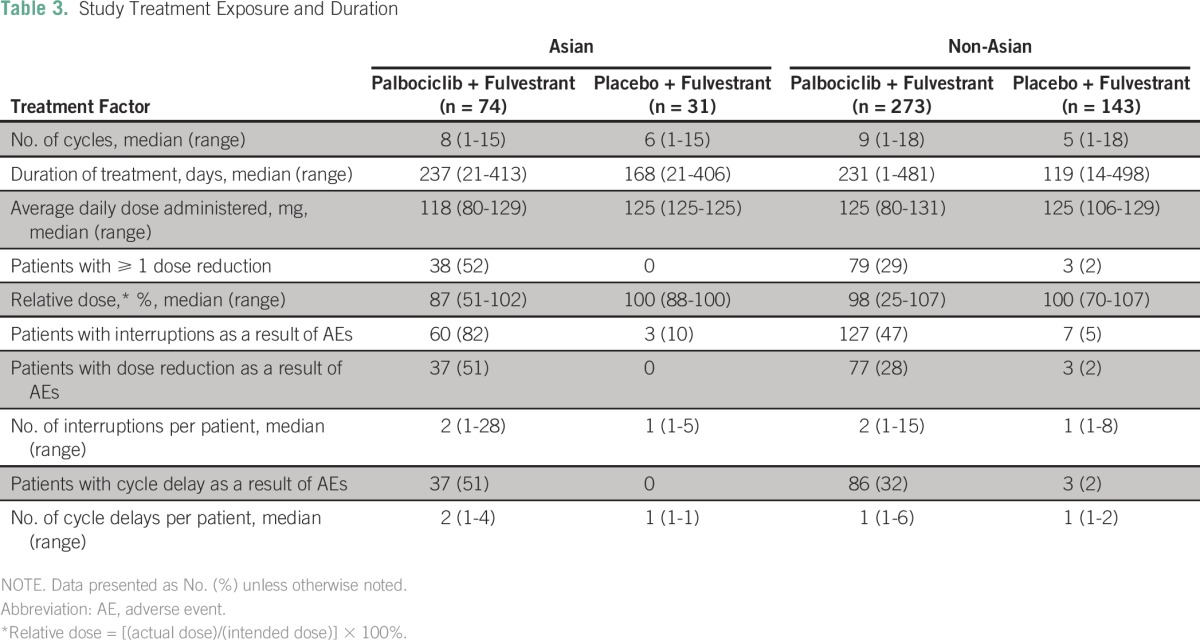
Study Treatment Exposure and Duration

**Table 4:**
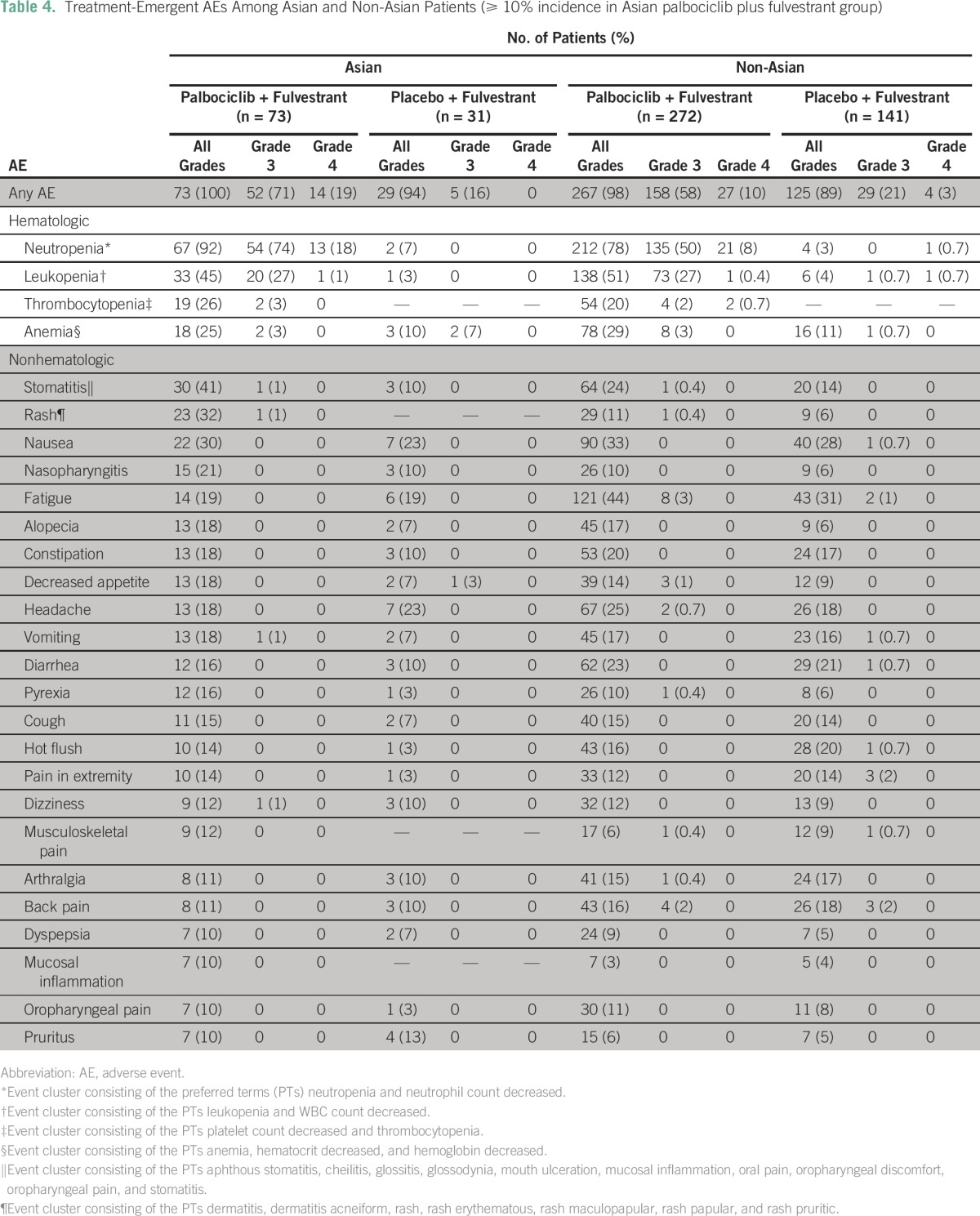
Treatment-Emergent AEs Among Asian and Non-Asian Patients (≥ 10% incidence in Asian palbociclib plus fulvestrant group)

The median number of treatment interruptions per patient was not different between Asians and non-Asians, regardless of treatment group. The number of cycle delays per patient was higher in Asians than non-Asians, regardless of treatment group. The median relative dose was lower in Asians than non-Asians in the palbociclib group and similar between Asians and non-Asians in the placebo group (Table 3). Fourteen non-Asian patients (5.1%) in the palbociclib arm and five non-Asian patients (3.5%) in the placebo arm discontinued palbociclib or placebo treatment because of an AE.

In Asians, the overall incidence of serious AEs was 14% (10 of 73 patients) in the palbociclib arm and 23% (seven of 31 patients) in the placebo arm (Appendix Table A1). In non-Asians, the incidence of serious AEs was 13% (34 of 272 patients) and 16% (23 of 141 patients) in the palbociclib and placebo arms, respectively. In the placebo plus fulvestrant group, the incidence of serious AEs in Asians (23%) was similar to the incidence in non-Asians (16%).

### PK Results

**Fig 3:**
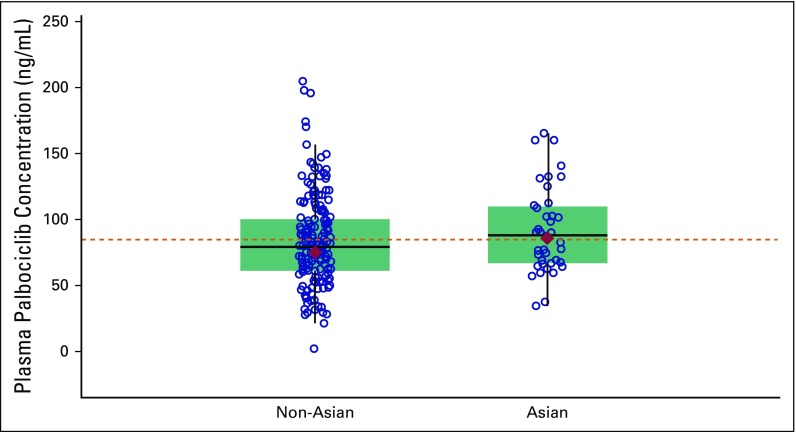
Plasma palbociclib within-patient mean steady-state trough concentration in Asian and non-Asian patients. Diamonds represent the subpopulation geometric mean values, and open circles represent individual patient values. The dashed line represents the arithmetic mean value of all data from all patients. The box plot provides median and 25% and 75% quartiles with whiskers to the last point within 1.5 times the interquartile range.

Comparison of the within-patient mean steady-state palbociclib trough concentrations in Asians and non-Asians demonstrated relative consistency in the central tendency and range of the observed values across subpopulations, indicating similar palbociclib exposure in these subpopulations (Fig 3). Geometric mean values of the within-patient mean steady-state palbociclib trough concentration values were similar for Asians and non-Asians (85.7 and 74.8 ng/mL, respectively). A population PK-pharmacodynamic (PD) analysis performed to assess the exposure-response relationship for neutropenia within PALOMA-3 showed that Asian race, baseline ALT level, and age were significant covariates on the baseline absolute neutrophil count (ANC) values. Asian race, lower baseline ALT level, and younger age were associated with lower baseline ANC values. Importantly, race was not found to be a covariate on any of the model PD response parameters. Generally, Asians in PALOMA-3 had a baseline ANC value that was 19% lower than non-Asians (Appendix Table A2).

### PROs

**Fig A1:**
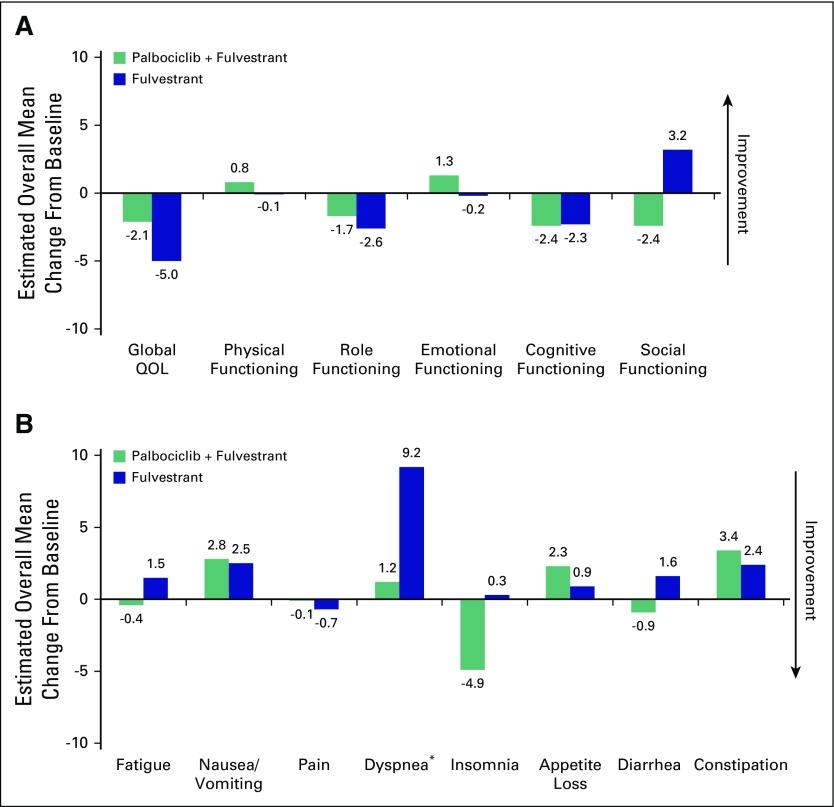
Overall mean (SE) change from baseline in patient-reported scores on the European Organisation for Research and Treatment of Cancer Quality of Life Questionnaire C30 of (A) global quality of life (QOL) and function and (B) symptoms in Asian patients. (*) *P* < .05 for palbociclib plus fulvestrant versus placebo plus fulvestrant.

Questionnaire completion rates were high at baseline and during treatment (from baseline to cycle 12, ≥ 90% of patients in each group completed all questions on the European Organisation for Research and Treatment of Cancer Quality of Life Questionnaire C30). In Asians, no significant deterioration from baseline in global QOL was observed within the palbociclib arm. Among the Asian subgroup in the study, no significant differences between treatment arms were observed for global QOL, functioning, pain, fatigue, or nausea and vomiting (Appendix Fig A1A). Significantly greater deterioration was observed in the placebo arm versus the palbociclib arm for dyspnea (score, 1.2 *v* 9.2, respectively; *P* < .05; Appendix Fig A1B).

## DISCUSSION

CDK4/6 inhibitors are now an integral part of the management of HR-positive/HER2-negative MBC.^17^ Palbociclib, the first-in-class CDK4/6 inhibitor approved for the treatment of HR-positive MBC, has shown impressive PFS improvement when combined with either an aromatase inhibitor^18^ or selective estrogen receptor downregulator^14^ in both patients who are endocrine sensitive and endocrine resistant. In the PALOMA-1 phase II study and PALOMA-2 phase III study of patients who had not previously received endocrine therapy, longer PFS was reported with palbociclib plus letrozole versus letrozole alone.^18,19^ Similarly, in the PALOMA-3 study, in patients who had previously received endocrine therapy, palbociclib plus fulvestrant resulted in longer PFS than fulvestrant alone.^14^ Palbociclib has been approved in the United States and has been used in more than 48,000 patients since February 2015.^20^ Palbociclib is also approved by regulatory authorities for advanced breast cancer in the following countries in Asia: Singapore, Malaysia, Macau, Hong Kong, and Korea. In many of these countries, palbociclib will be reviewed by health technology agencies, payers, or both. The positive clinical value of palbociclib in Asian patients should be considered alongside the economic implications.

Substantial clinical experience has been accumulated in white patients. Although few Asians were enrolled onto the PALOMA-1 study,^21^ 21% of patients in the palbociclib arm and 18% of patients in the fulvestrant arm in PALOMA-3 were Asian.^14^ This study adds to the limited body of literature assessing a CDK4/6 inhibitor in Asians and represents the largest patient experience with palbociclib in Asians. The present findings show that palbociclib plus fulvestrant improved PFS in Asians with HR-positive/HER2-negative MBC who experienced progression on prior endocrine therapy and that the safety profile of palbociclib plus fulvestrant in Asians was generally consistent with that observed in non-Asians. Together, these findings suggest that palbociclib is beneficial in patients who have not previously received endocrine therapy and in Asians and non-Asians who experienced relapse or progression during prior endocrine therapy.

Differences in racial background can be associated with variable efficacy outcomes and safety profiles.^22^ As a result of genetic variations in an enzyme responsible for doxorubicin metabolism,^23^ Asians have been shown to be more susceptible to myelosuppression induced by doxorubicin compared with whites.^22^ In addition, a higher incidence of febrile neutropenia with docetaxel has been reported in Asians compared with whites.^22^ Genetic differences associated with race also can lead to differences in treatment response and efficacy. In Koreans with MBC, CYP2D6*10/*10 genetic polymorphisms have been associated with reduced plasma concentrations of the tamoxifen active metabolites endoxifen and 4-hydroxytamoxifen, as well as reduced clinical benefit (complete response, partial response, or stable disease ≥ 24 weeks) and significantly shorter median time to progression (*P* = .0032).^24^ These racial variations highlight the importance of evaluating the efficacy and safety of cancer medications within the Asian population.

Similar to findings from the present analysis, the most common AEs reported in the PALOMA-1 study with palbociclib were neutropenia and leukopenia.^14,18^ In the PALOMA-3 study, nonhematologic AEs were predominantly mild or moderate in severity. Moreover, an important difference of treatment exposure was observed between Asians and non-Asians in the palbociclib arm, with higher percentages of Asians experiencing dose interruptions, dose reductions, and cycle delays than non-Asians. Interestingly, the rates of grade 3 and grade 4 neutropenia were modestly higher in Asians than non-Asians. Because palbociclib exposure was similar in Asians and non-Asians, the increased rates of neutropenia cannot be explained by differential drug exposure across racial subgroups. Asian race, lower baseline ALT, and younger age were all predictors of a lower baseline ANC value. The Asians in PALOMA-3, on average, were younger and had a lower baseline ALT than the non-Asians, thus compounding effects of the covariates. Overall, in the PALOMA-3 patient population, a typical Asian patient (52 years old at enrollment with a baseline ALT of 17 U/L) had a baseline ANC value 19% lower than a typical non-Asian patient (58 years old at enrollment with a baseline ALT of 21 U/L), which may partially explain the higher rate of neutropenia observed in Asians. Importantly, race was not demonstrated to be a covariate on any of the PD response parameters, suggesting that there was no increased sensitivity to palbociclib-induced neutropenia in Asians.

MBC in premenopausal women is not well studied because clinical trials often exclude this patient population. One phase II study of 73 patients with HR-positive MBC showed that the efficacy of first-line therapy with letrozole plus goserelin in premenopausal patients was comparable with the efficacy of letrozole alone in postmenopausal patients^25^; these findings support additional research into assessing the efficacy of other treatments in combination with goserelin in premenopausal patients with breast cancer. MBC in premenopausal women is rare in the Western world; however, higher incidences are seen in Asian countries and in developing countries such as Mexico, Latin America, and Egypt, where breast cancer is more common in younger women and is frequently diagnosed at later stages as a result of suboptimal access to health care.^3,4,26-29^ Palbociclib plus fulvestrant improved PFS in both premenopausal and postmenopausal Asians in PALOMA-3. Because of the small number of patients in this cohort, no formal statistical analysis could be performed. Nevertheless, palbociclib plus fulvestrant in addition to an LHRH agonist could be a reasonable treatment option for younger patients with breast cancer who are premenopausal, including Asian patients.

Assessing PROs is important to comprehensively define the risk-benefit profile of treatments. In the current study, Asians in the palbociclib group maintained good QOL throughout the study, which is important in establishing the benefit-risk profile of combination therapy.

In conclusion, as observed in the full study population, PFS was longer in Asians with HR-positive/HER2-negative MBC who received palbociclib plus fulvestrant versus those who received placebo plus fulvestrant. Furthermore, QOL was maintained in Asians who received palbociclib. The safety profile of palbociclib was consistent with that previously reported and was similar in Asians and non-Asians. The protocol-defined dosing modification instructions for palbociclib, including adjusting dose on the basis of individual tolerability, enabled Asians to avoid discontinuation from the study as a result of an AE, allowing them to stay on treatment as long as non-Asians and thus maintain the same efficacy benefit from combination therapy. Overall, palbociclib plus fulvestrant seems to be a reasonable treatment option in Asians with HR-positive/HER2-negative MBC that has progressed on prior endocrine therapy.

## Figures and Tables

**Table A1:**
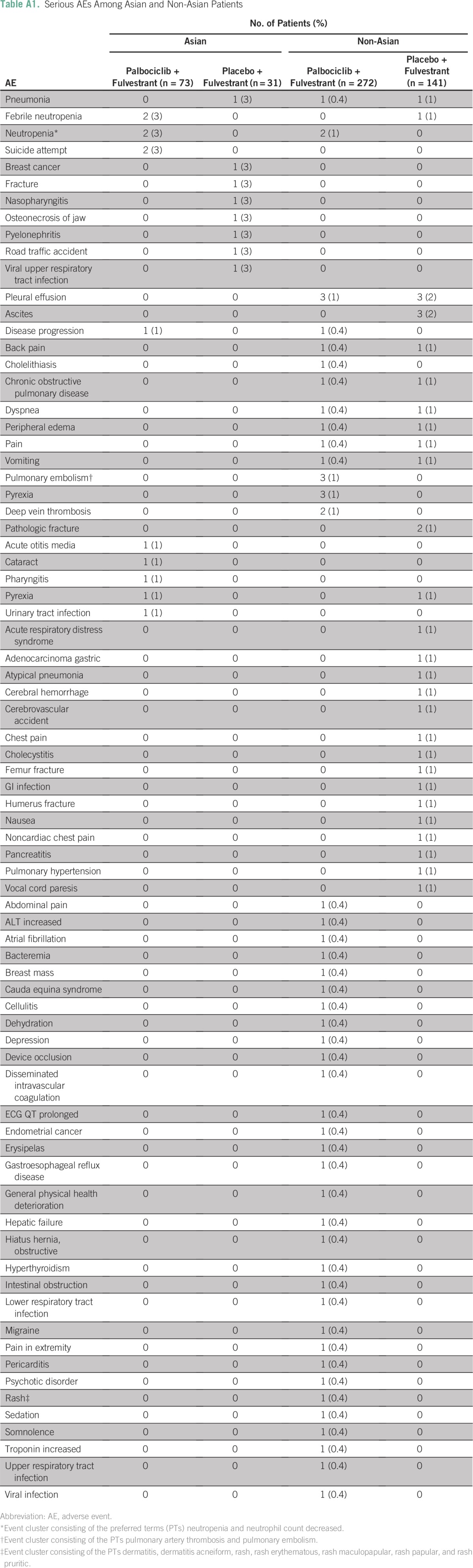
Serious AEs Among Asian and Non-Asian Patients

**Table A2:**
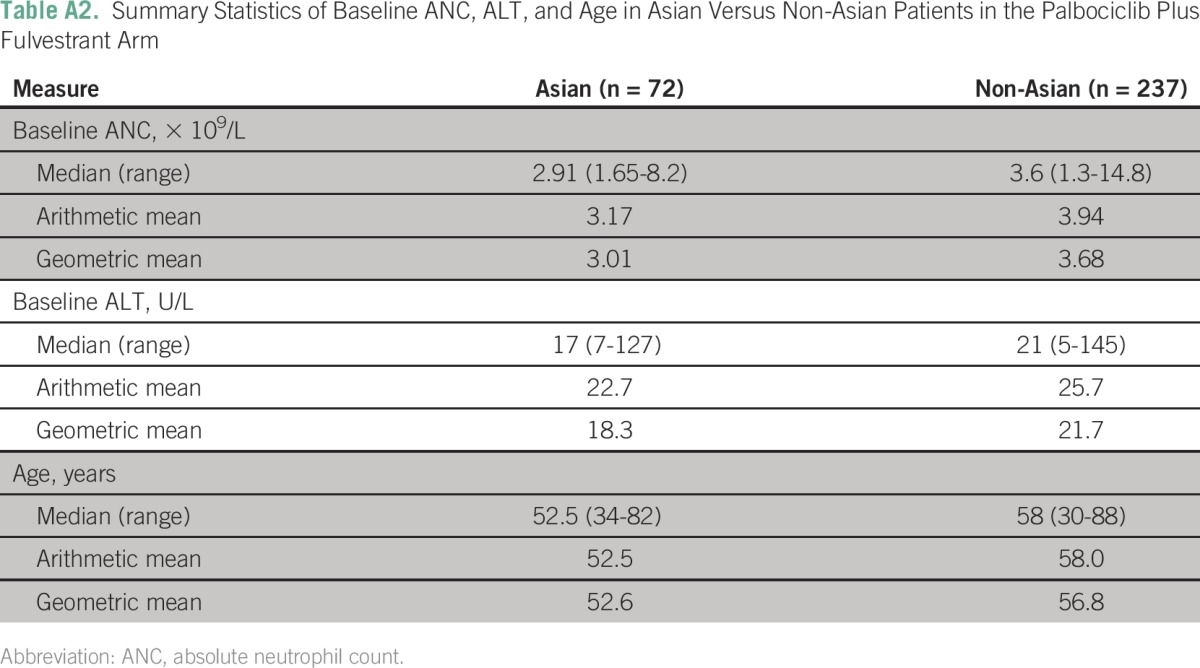
Summary Statistics of Baseline ANC, ALT, and Age in Asian Versus Non-Asian Patients in the Palbociclib Plus Fulvestrant Arm
